# Type I Interferon at the Interface of Antiviral Immunity and Immune Regulation: The Curious Case of HIV-1

**DOI:** 10.1155/2013/580968

**Published:** 2013-12-22

**Authors:** Adriano Boasso

**Affiliations:** Immunology Section, Chelsea and Westminster Hospital, 369 Fulham Road, London SW10 9NH, UK

## Abstract

Type I interferon (IFN-I) play a critical role in the innate immune response against viral infections. They actively participate in antiviral immunity by inducing molecular mechanisms of viral restriction and by limiting the spread of the infection, but they also orchestrate the initial phases of the adaptive immune response and influence the quality of T cell immunity. During infection with the human immunodeficiency virus type 1 (HIV-1), the production of and response to IFN-I may be severely altered by the lymphotropic nature of the virus. In this review I consider the different aspects of virus sensing, IFN-I production, signalling, and effects on target cells, with a particular focus on the alterations observed following HIV-1 infection.

## 1. Introduction

Interferons (IFN) are a heterogeneous class of soluble immune mediators which were originally defined by their ability to interfere with the replication of diverse types of viruses *in vitro* and *in vivo*. Human type I IFN (IFN-I) include IFN-*α* (14 genes, resulting in more than 22 products) and IFN-*β*, both encoded by genes clustered on chromosome 9, probably generated as a result of gene duplication events, as epitomized by the presence of multiple pseudogenes [1]. IFN-*ε*, IFN-*κ*, IFN-*τ*, and IFN-*ω* have also been described in mammals as members of the IFN-I family. This review is focused on the literature covering the regulation and the function of IFN-*α* and IFN-*β*.

IFN-I are produced in large quantities in response to viral infections and are generally regarded as a key bridging mechanism between innate and adaptive immune responses, exerting both antiviral activity, and immunostimulatory functions, such as promoting antigen-presenting cell maturation and molding T helper cell responses [2–8]. However, the notion that IFN-I serve only as effector immune mechanisms may need to be revised based on accumulating evidence which highlights the potent immunoregulatory ability of these molecules. During chronic viral infections, the beneficial antiviral and immunostimulatory effects of IFN-I may be overruled by the prolonged stimulation of IFN-I-induced cytostatic and proapoptotic mechanisms.

Infection by the human immunodeficiency virus type 1 (HIV-1) may represent an extreme example of how chronic IFN-I production progressively undermines the development of efficient long-term antiviral immunity [9, 10].

Different cells have the potential to produce IFN-I and the mechanism by which viral insults are sensed varies depending on the cell type, as does the potency of the IFN-I-producing response. In the case of HIV-1, most studies have focussed on plasmacytoid dendritic cells (pDC) as the main producers of IFN-I following viral exposure [9, 11, 12]. However, recent evidence indicates that the source and dynamics of IFN-I production may change during the course of infection, and pDC may be replaced by myeloid dendritic cells (mDC) and monocytes/macrophages as the main source of IFN-I during the transition from acute to chronic infection [13].

Based on the molecular mechanisms of viral sensing involved in HIV-1 recognition by different cells (1), the evidence available on IFN-I production by different cell types during the acute and chronic phases of infection (2) and the combination of antiviral and immunomodulatory activity of IFN-I (3), it is possible to speculate that IFN-I may serve different purposes during different stages of chronic infections, and that the dysregulation of IFN-I production during HIV-1 infection may contribute to progressive immunodeficiency.

## 2. Viral Sensing by Pattern Recognition Receptors

Innate sensing of virus-related pathogen associated molecular patterns (PAMP) relies primarily on recognition of viral nucleic acids by toll-like receptors (TLR) in the endosomes of specialised immune cells, or by cytoplasmic sensors widely expressed by different cell types and not restricted to immune cells [14–19].

HIV-1 interacts with and infects cells expressing CD4, which is engaged by the viral envelope glycoprotein gp120. Thus, IFN-I production during HIV-1 infection is mainly sustained by immune cells which are either exposed to or infected by HIV-1.

### 2.1. Toll Like Receptors

Toll-like receptors are a family of pattern recognition receptors (PRR) preferentially expressed by cells which participate in the innate immune responses against invading pathogens [14–16]. At least four TLR have the potential to sense viral nucleic acids in humans. Signalling via TLR3, TLR7, and TLR8 is triggered by viral RNA, whereas TLR9 recognizes unmethylated CpG-rich DNA sequences [15, 20]. Although RNA-sensing TLR have the potential to recognize HIV-1, only TLR7-expressing pDC appear to respond promptly to viral exposure. Conversely, the available evidence either excludes or is inconclusive regarding the ability of HIV-1 to directly activate cells expressing TLR8 or TLR3, such as mDC and MΦ, but also microglia and astrocytes within the central nervous system [12, 21–23].

The ability of pDC to respond to HIV-1 stimulation appears to be mainly dependent on TLR7 signalling [24], although a role for TLR9 cannot be excluded [12]. The HIV-1 viral cycle includes the formation of a DNA/RNA heterodimer and a double stranded DNA proviral DNA, which represent potential ligands for TLR9 via CpG-rich DNA regions. Partial reverse transcription may occur already in the virion, due to the packaging of complexes formed by reverse transcriptase, viral RNA genome and transfer RNA primer [25–27]. However, the reverse transcription process is completed in the cytoplasmic environment [25, 27], secluded from endosomal TLR9. Whether fragment of DNA of viral origin included in the virion engulfed by pDC and processed via the endosomal pathway can trigger TLR9-mediated responses remains untested.

Engagement of TLR7/9 results in the activation of a complex network of transduction signalling pathways in pDC. The first step required for TLR7/9 signal transduction is the recruitment of the adaptor molecule myeloid differentiation primary-response gene 88 (MyD88), which in turn associates with other adaptor and signalling molecules to form a complex comprised of TNF-receptor-associated factor (TRAF)6, Bruton's tyrosine kinase (BTK), IL-1R-associated kinase (IRAK)4 and IRAK1 [20, 28, 29]. The signalling complex catalyzes the activation of different intracellular pathways.

The pathway mediated by the interferon regulatory factor (IRF)7 is directly associated with activation of IFN-*α* and IFN-*β* genes following nuclear translocation of IRF7 [30]. Signalling through the IRF7 pathway is dependent on the activation of phosphatidylinositol 3-kinase (PI3K)-*δ* [31]. IFN-*α*/*β* secreted during this early phase may act in an autocrine manner through the dimeric type I IFN receptor (IFNAR) and stimulate *de novo* production of IRF7, further stimulating IFN-I secretion in a potent positive feedback loop of IFN-I production [28]. The IRF7-mediated signalling pathway is therefore responsible for the differentiation of pDC into efficient IFN-I producing cells (IPC). PI3K and IRF7 signalling is not required for the production of tumor necrosis factor (TNF)-*α*, interleukin (IL)-6, and the chemokines CXCL10 and CCL3, which rely on the canonical nuclear factor (NF)-*κ*B pathway (Rel-A: p50 dimer) which in turn requires mitogen-activated protein kinase p38 (p38-MAPK) activity [32]. The same pathway is responsible for promoting the expression of the costimulatory molecules CD80 and CD86, which are conditions necessary for the maturation of pDC into fully competent antigen presenting cells (APC) [32]. TLR7/9 engagement also promotes upregulation of the immunoregulatory enzyme indoleamine (2,3)-dioxygenase (IDO) in both murine and human pDC [33–36]. In murine models, IDO regulation via TLR7/9 occurs following activation of the noncanonical NF-*κ*B pathway (Rel-B: p52 complex) [37], and non-canonical NF-*κ*B is also required to support IFN-*α* production [38].

IRF7 and NF-*κ*B are not simultaneously activated following TLR7/9 engagement, and the pathway which dominates the signalling cascade is determined by the intracellular compartment where TLR7/9 engagement occurs [39].

### 2.2. Cytoplasmic RNA Sensors

TLR represent powerful mechanisms for viral recognition, but their expression is limited to immune cells and the restriction to endosomal compartment prevents them from detecting viruses which have reached the cytoplasmic environment. Retinoic acid-inducible gene 1 (RIG-I)-like receptors (RLR) are a family of PRR broadly expressed among different human cells, and confer the ability to sense virus-derived RNA within the cytoplasm [15, 17, 18]. RLR include RIG-I, melanoma differentiation factor (MDA)5, and laboratory of genetics and physiology (LGP)2 protein, all of which are potently upregulated by IFN-I [40]. Studies conducted in transgenic mice suggest that different RLR are critical in the recognition of and IFN-I response against different viruses [18]. However, it remains unclear how RLR distinguish viral RNA from cellular RNA, and therefore trigger IFN-I production only in infected cells. The specificity for exogenous RNA may be dependent on the recognition of secondary structures which are common in viral RNA genomes [18]. RLR signal transduction occurs through binding of the receptor to the mitochondrial adaptor IFN-*β* promoter stimulator (IPS)-1 and formation of a signalling complex leading to the activation of the kinases TNF receptor-associated factor (TRAF)-associated NF-*κ*B activator (TANK) binding kinase (TBK)1-IK*β* kinase (IKK)*ε* [40]. The TBK1-IKK*ε* is then responsible for both IFN-I production via IRF3 and IRF7 and for NF-kB activation [40].

Berg and colleagues demonstrated that genomic HIV RNA can trigger inflammatory responses in human peripheral blood mononuclear cells (PBMC) via RIG-I recognition, leading to production of the interleukin (IL)-6, TNF-*α*, IFN-*α*, and IFN-*β* [41]. The cellular sources of the proinflammatory cytokines were not investigated in this study. It is plausible that, during HIV-1 infection *in vivo*, all cells susceptible of infection have the potential to respond to HIV-1 genomic RNA via RIG-I sensing. However, the nature of the responses may differ depending on the target cells, and inflammatory responses may be differentially mediated by DC and monocyte/macrophages compared to CD4 T lymphocytes.

### 2.3. Cytoplasmic DNA Sensors

The presence of a RNA-DNA hybrid during reverse transcription of the HIV-1 RNA genome into dsDNA suggests that cytoplasmic recognition of the proviral DNA may occur before its translocation to the nucleus and integration into the host genome.

The cytosol of eukaryotic cells is rich in enzymes with DNase activity, which prevent accumulation of DNA in the cytoplasm [42]. However, when the preventive measures exerted by cytoplasmic DNases fail, DNA of viral origin may accumulate in the cytoplasm. Double stranded DNA is a potent immune stimulator when present in the cytosol of target cells [43, 44], and recent evidence indicates that single-stranded DNA with specific signatures, such as AT-rich regions, is also a strong activator of immune responses [45]. Sensing of cytoplasmic DNA triggers a cascade of signal transduction orchestrated by the adaptor molecule stimulator of interferon genes (STING) [46–49], leading to the production of proinflammatory and antiviral cytokines, including IFN-I [48]. STING engages TBK1 to cause IRF3 activation, and the STING-TBK1-IRF3 signaling axis is critical for IFN-I induction by cytosolic DNA [50].

Recent evidence indicates that cytosolic HIV-1-derived dsDNA or RNA/DNA heterodimers may trigger innate immune responses. Gao and colleagues have used both the monocytic cell line THP1 and primary human monocyte-derived macrophages and dendritic cells to show that HIV infection induces the production of cyclic guanosine monophosphate-adenosine monophosphate (cGAMP), which binds to and activates STING resulting in the production of IFN-I [51]. The production of IFN-*β* was strictly dependent on reverse transcription, indicating that HIV-1 DNA acts as the initial trigger for IFN-I production [51]. Furthermore, Jakobsen and colleagues have shown that ssDNA generated from HIV-1 proviral genome is a potent activator of IFN-I in primary human monocyte-derived macrophages [52]. Single stranded HIV-1 DNA engages the IFN-inducible protein 16 (IFI16) in the cytoplasm of macrophages, leading to the activation of the STING-TBK1-IRF3 pathway [52].

## 3. Cellular Sources of IFN-I during HIV-1 Infection

### 3.1. Plasmacytoid Dendritic Cells

Plasmacytoid DC are the most potent producers of IFN-I in response to viral infections [16, 53, 54]. Expression of endosomal TLR7 and TLR9 allows pDC to respond to both RNA and DNA viruses which are engulfed and trafficked into the endosomal pathway. Upon TLR7/9 engagement, pDC can mature into antigen-presenting cells (APC) or IFN-I-producing cells (IPC), and the prevalence of one pathway over the other largely depends on the intracellular locale in which the TLR ligand triggers its receptor [23, 39]. Thus, the engagement of TLR7/9 within the early endosomes causes strong activation of the IRF7 pathway via an IFN-*α*/*β*-dependent positive feedback, resulting in the production of high quantities of IFN-*α* and pDC differentiation into IFN-I-producing cells (IPC) [39]. Conversely, if the TLR ligand is trafficked to the lysosomes or late endosomes, signal transduction via the NF-*κ*B pathway is favored, resulting in the upregulation of costimulatory molecules and maturation into APC [39]. O'Brien and colleagues reported that HIV-1 is preferentially trafficked to the early endosomes of pDC, which in turn promotes a persistently activated IPC status, combined with partial or incomplete maturation [23].

The binding of the HIV-1 envelope glycoprotein gp120 to CD4, but not to the coreceptors CCR5 or CXCR4 is required for virion engulfment and subsequent activation of pDC via TLR7 engagement [12, 55]. The interaction between gp120 and CD4 is stabilized by accessory interactions involving cellular adhesion molecules on both the cell membrane and viral envelope, allowing the formation of a stable binding interface between HIV-1 and the target cells [56–58]. Thus, the envelope of newly formed HIV-1 virions incorporates cellular proteins derived from the host cell of origin [56, 57, 59]; these cell-derived proteins contribute to virus-cell interactions and may modulate the dynamics of infection of target cells and uptake by endocytotic cells [57–60]. In addition, the HIV-1 envelope is not homogenous, but rather organized in a functional substructure enriched in tightly packed cholesterol, similar to the lipid rafts described in eukaryotic cells, including human leukocytes [9, 60, 61]. The organization of a functional virion-associated lipid raft is required to confer HIV-1 the ability to induce potent IFN-I production via pDC stimulation [9]. Thus, the efficiency of HIV-1 uptake by pDC and the potency of IFN-I induction are strongly reduced if envelope-associated cholesterol is withdrawn by chemical treatment [9]. However, cholesterol depleted HIV-1 partially retains the ability to promote pDC maturation into APC [9], suggesting that the integrity of the virion-associated lipid raft may not only reduce the efficiency of HIV-1 uptake, but also modify the dynamics of HIV-1 trafficking and subsequent TLR signalling in favour NF-*κ*B-mediated APC maturation rather than IRF7-dependent IFN-*α* production.

Activation of pDC may occur within hours from exposure to HIV-1 *in vivo*. Intravaginal infection of rhesus macaques caused rapid accumulation of activated pDC at the mucosal site of infection, which resulted in macrophage inflammatory protein (MIP)-3*α*-mediated chemoattraction and infection of CCR5-expressing CD4 T cells, contributing to the spread of the infection to secondary lymphoid tissues [62]. Lubong Sabado and colleagues reported that pDC relocate to lymphoid tissues already during primary HIV-1 infection, a condition which persists throughout the course of disease [63]. However, reports on IFN-*α* secretion by pDC in lymphoid tissues during chronic HIV-1 infection showed contrasting results. For example, although IFN-*α* and upregulation of IFN-stimulated genes (ISG) has been reported in tissues from HIV+ patients [64, 65], Nascimbeni and colleagues showed that pDC in the spleen of HIV-infected patients have an immature phenotype and do not contribute to the increased IFN-*α* production [66]. The state of partial or incomplete pDC maturation is confirmed in the study by Benlahrech and colleagues, who have recently shown that expression of the immunoglobulin-like transcript (ILT) 7, a regulatory receptor expressed by immature circulating pDC but not partially differentiated cells [36], is reduced in pDC from HIV-infected patients when viral replication is not efficiently controlled by therapy [67].

Recent evidence suggests that pDC may be the predominant source of IFN-I only during the initial phases of acute infection, whereas mDC and macrophages become important producers of IFN-*α* when the course of infection transitions to the early chronic phase [13]. Both mDC and monocyte/macrophages express TLR8, which has the potential to recognize viral RNA [68, 69]. However, several studies have shown that *in vitro* maturation of mDC and monocyte in presence of HIV-1 is a bystander effect occurring in response to cytokines produced by HIV-1-activated pDC, such as IFN-*α* and TNF-*α* [12, 21–23]. In addition, even in conditions in which TLR8 engagement occurs, mDC do not respond by secreting IFN-I, but rather mature into interleukin (IL)-12-secreting APC [68, 69]. Thus, IFN-I production by mDC and macrophages during the post-acute and chronic phases of HIV-1 infection may depend on molecular pathways other than sensing of extracellular viral particles virus via endosomal TLR.

### 3.2. Monocytes/Macrophages and Myeloid Dendritic Cells

Endosomal TLR allow recognition of nucleic acids from viruses engulfed by specialized cells, but may not sense viral genomes which have gained access to the cytoplasm. Intracellular RLR and DNA sensors are triggered by viral genome in the cytoplasm and induce the production of antiviral and immunostimulatory cytokines, such as IFN-*α* and TNF-*α* [18]. RLR are expressed by most cell types and their triggering requires that the viral genome has reached the cytoplasm, a condition which is also necessary to achieve productive infection of the target cell. Conversely, TLR7/9-mediated pDC responses would not be triggered during a productive infectious cycle, due to the segregation of the receptors in the endosomal compartment. This may represent a critical difference between IFN-I responses orchestrated by uninfected pDC during the early phases of viral exposure and IFN-I production by productively infected cells during later stages of infection.

This transition from a pDC-mediated to an apparently pDC-independent IFN-I response has been recently described in the HIV-1 simian model of simian immunodeficiency infection (SIV) of Rhesus macaques by Kader and colleagues [13]. Monocyte derived macrophages have been shown to respond to HIV-1 proviral DNA via intracellular DNA sensors [51, 52], and genomic HIV-1 RNA activates innate immune responses in peripheral mononuclear cells via RLR [41]. The question can be raised as to whether the main source of IFN-I changes during HIV-1 infection in relation to a shift in the distribution of productively infected cells among different cell types. Dendritic cells may act as catalysts for the infection of CD4 T cells via cell-cell transfer [70, 71], but activated CCR5+ CD4 T cells are the main target for infection and the main source of viral replication throughout the acute phase [72, 73]. However, the number of CCR5+ T cells decreases progressively during acute infection both in the periphery and in lymphoid tissues, likely due to a combination of viral cytotoxicity, activation-induced apoptosis and immune-dependent killing mediated by newly activated HIV-1-specific cytotoxic CD8 T lymphocytes (CTL) [72–74]. Thus, as the pool of CCR5+ CD4 T cells decreases, CD4-expressing mDC and monocyte/macrophages may become increasingly more important as targets for infection, and therefore more susceptible to activation via cytoplasmic RLR.

The critical questions that remain unanswered is whether the prolonged IFN-I response observed during pathogenic HIV-1/SIV infection plays a determinant role in viral immunopathogenesis and whether pDC-mediated acute responses or chronic IFN-I responses mediated by non-pDC subsets are potentially valuable targets for immunotherapeutic or curative interventions.

### 3.3. Other Cellular Sources of IFN-I

The broad expression of RLR and cytosolic DNA sensors in numerous cell types and in different tissues [18] suggests that any cell susceptible to HIV-1 infection, and therefore to cytoplasmic exposure of viral RNA, has the potential to secrete IFN-*α* during HIV-1 infection. Nonetheless, evidence about IFN-*α* production by cells other than DC and monocytes is scattered and largely inconclusive about its relevance for systemic immune activation and immunopathogenesis. However, IFN-*α*-producing cell subsets which are confided to specific anatomic locations may contribute to inflammatory processes within non lymphoid tissues and organs, partially accounting for the development of HIV-1-associated non-communicable comorbidities (NCCM). An increasing body of evidence suggests a causative link between chronic inflammation and some manifestations of NCCM. One example is that of neurological complications associated with neuroinflammation, which are observed even in patients in whom viral replication in controlled by combination antiretroviral therapy (cART) [75–77]. Thus, microglial activation and an IFN-driven gene regulation signature are characteristic of HIV-associated neurological symptoms, such as cognitive impairment and depressive disorder [75, 76, 78, 79]. It is noteworthy that neurologic disease associated with infection with another human lymphotropic retrovirus, the human T-lymphotropic virus type 1 (HTLV-1), is also associated with an IFN-dominated inflammatory profile. Thus, Tattermusch and colleagues described an IFN-inducible signature in HTLV-1-associated myelopathy/tropical spastic paraparesis (HAM/TSP) [80, 81]. It is still unclear whether peripheral sources of IFN-I, such as DC or macrophages are the sole responsible for the neuropathologic effects or whether astrocytes and microglial cells are directly involved in the production of IFN-I during the inflammatory response. Whether IFN-I production by these cells plays a role also in the immunopathogenesis of HIV-1 infection is yet to be determined.

## 4. Effect of IFN-I on Target Cells

Type I IFN are the most potent natural mediators of antiviral activity in humans. The combination of proapoptotic and cytostatic activity, as well as the direct induction of intracellular viral restriction factors contribute to generate a cellular environment which is unsuitable for viral replication [1, 3, 82, 83]. The replication of HIV-1 is efficiently inhibited by IFN-I *in vitro*, but the potential of IFN-I as antiretroviral agents during chronic HIV-1 infection *in vivo *is dubious [84–89].

IFN-I also contribute to the maturation of APC and exert a modulatory effect on different T cell subsets, therefore posing the foundations for promoting antigen-specific adaptive immune responses and shaping them according to the invading pathogen [1, 6, 7, 53, 82, 90].

### 4.1. IFN-*α* Receptor Complex and Signalling

The cellular effects of IFN-I are mediated by the engagement of a common receptor complex, the IFN-*α* receptor complex (IFNAR), which is expressed at different levels on the surface of virtually all human cells. The IFNAR complex is a heterodimer of the two subunits IFNAR1 and IFNAR2. Human IFNAR1 comprises an extracellular domain, a transmembrane region and an intracellular domain of 100 amino acid residues. Three different forms of IFNAR2 have been described: the full length receptor chain which includes a 250 residues cytoplasmic portion, an alternative form with a shorted intracellular portion (67 residues) [91, 92] and a soluble form lacking the transmembrane and cytoplasmic portions [91]. Human IFNAR2 binds all human IFN-I with higher affinity than IFNAR1, but the affinity of the IFNAR heterodimer for most human IFN-I is 10 fold higher than IFNAR2 alone [91, 93, 94].

The cytoplasmic domains of IFNAR1 and IFNAR2 are associated with the tyrosine kinase (TYK)-2 and janus kinase (JAK)-1, respectively [91]. Upon IFN-I binding to the IFNAR complex, the tyrosine kinases are activated and orchestrate the signal transduction machinery [95]. TYK-2 and JAK-1 phosphorylate tyrosine residues on the IFNAR, and phosphorylated tyrosines act as docking sites for the src-homology-2 (SH2) domains of signal transducer and activator of transcription (STAT) proteins, which are then targeted for phosphorylation [96, 97]. STAT1, STAT2, STAT3, and STAT5 are expressed and activated by IFN-I in most cell types, whereas IFN-I-induced activation of STAT4 and STAT6 is limited to lymphocytes [96, 97].

STAT2 is recruited to the IFNAR1 cytoplasmic domain and is phosphorylated by TYK-2, serving as a lure for STAT1 [98], which is also phosphorylated at tyrosine 701. The STAT1-STAT2 heterodimer associates with the DNA binding protein interferon regulatory factor 9 (IRF9) to form a signalling complex called IFN-stimulated gene factor-3 (ISGF3). The ISGF3 complex translocates to the nucleus where it promotes the transcription of IFN-I-stimulated genes (ISG) by interacting with the IFN-stimulated response elements (ISRE), usually situated within 200 base pairs of the transcription start site. An additional phosphorilation of STAT1 at serine 727, mediated by protein kinase C-*δ* (PKC-*δ*), is essential for efficient transcriptional activity [1, 98]. In addition, STAT1 homodimers can bind to IFN-*γ*-activated site (GAS) in the promoter region of IFN-I-stimulated genes [99, 100]. Thus, while IFN-I can activate genes containing either ISRE or GAS promoter elements, IFN-*γ* is unable to induce the formation of ISGF3 and activate genes via ISRE [101]. Signalling via STAT1-STAT2 is responsible for some of the most commonly observed effects of IFN-I during chronic HIV-1 infection, including the activation of proapoptotic and cytostatic genes, viral restriction factors and immunomodulatory effects [102–104].

Type I IFN also triggers phosphorylation of STAT3 [105], promoting its dimerization and migration to the nucleus. STAT3 homodimers activate the transcription of genes containing the enhancer sequence STAT3-binding element (SBE). Despite sharing high sequence similarity with STAT1, STAT3 activation induces a gene expression profile distinct from that dependent on STAT1-STAT2 [106], including genes of the B cell lymphoma (BCL) family and the oncogene MYC, which promote proliferation and antagonize apoptosis [107, 108]. Furthermore, STAT3 is responsible for the suppression of inflammatory responses via IL-10, which directly counteracts STAT1 activity [109].

IFN-*β* also activates the signal transduction pathway mediated by JAK1 and phosphoinositide 3-kinase (PI3K), leading to the expression of genes regulated by cyclic adenosine monophosphate (cAMP) via the nuclear translocation of cAMP-responsive-element (CRE) binding protein (CREB). Similar to the STAT3-mediated pathway, PI3K signalling can also result in IL-10 production by DC [110]. However, the two pahways appear to be independent from each other. The mammalian target of rapamycin (mTOR) is activated downstream of the PI3K signalling cascade [111]. Activation of mTOR is independent of STAT signalling and does not modify the gene expression profile, but modulates mRNA translation by regulating the activation of p70 S6 kinase and the phosphorylation of the ribosomal protein S6 [112, 113].

The signalling pathway mediated by p38 mitogen-activated protein kinases (p38-MAPK) and extracellular signal-regulated kinase (ERK) 1 or ERK2 is also activated by IFN-I. Thus, genes which contain ISRE and GAS elements in their promoter region can be upregulated via a STAT-independent pathway mediated by p38-MAPK [114–116], and the cytostatic and antiviral effects of IFN-I are dependent on intact p38-MAPK signalling machinery [117–120].

It is reasonable to expect that different IFN-I signal transduction pathways are activated during HIV-1 infection. However, the STAT1-STAT2 pathway is arguably the best described in the setting of IFN-I production during HIV-1 infection. The activation of STAT proteins by HIV-1 was described in different experimental conditions involving HIV-1-induced IFN-I production, productive HIV-1 infection of target cells or simple exposure to viral proteins. Thus, *in vitro* exposure of cell lines or primary leukocytes to whole HIV-1 virions was repeatedly shown to activate STAT-dependent pathways in CD4 T cells and cells of the monocytic lineage [55, 121, 122]. Interestingly, Renga and colleagues reported that exposure of monocytic cell lines to the HIV-1 matrix protein p17 was sufficient to activate the JAK1/STAT1 axis and cause upregulation of STAT1 sensitive genes [123], suggesting that STAT1 activation during HIV-1 infection may also occur independent of IFN-I production. Simian immunodeficiency virus (SIV) infection of nonhuman primates *in vivo* resulted in upregulation of gene expression for STAT1, STAT2 and IRF9 during the acute phase of infection, which was protracted through the early chronic phase only in disease susceptible primate species, not in disease resistant African species [124, 125]. Although signalling via the ISGF3 complex is regulated by post-transcriptional modification of STAT proteins, not by modulation of STAT and IRF9 gene expression, the observed increases in the expression of these molecules in SIV-infected macaques [124, 125] is indicative of enhanced activity of the IFN-I/ISGF3 axis.

The central role played by PI3K in multiple signal transduction pathways renders it difficult to distinguish its participation in mediating the effects of IFN-I from other biochemical, cellular and molecular events occurring during HIV-1 infection. The contribution of PI3K in neuroinflammation associated with HIV-1 infection has been described [126–128], but the role of Pi3K/mTOR in HIV-1 immunopathogenesis remains unclear.

Activation of the p38-MAPK pathway during HIV-1 infection has been described by several groups [129–139]. However, studies on the p38-MAPK/ERK pathway have generally focused on the direct effect of HIV-1 gp120 singalling via engagement of CD4 or the coreceptors CCR5 and CXCR4 [129, 136–139], rather than IFN-I production. In particular, activation of p38-MAPK/ERK has been studied in reference to its role in modulating cell activation and HIV-1 replication [129–135].

### 4.2. Expression Pattern of IFNAR

Type I IFN are generally considered to exert their biologic effect on virtually all human cells. This broad and noncell specific activity reflects the prominent innate antiviral function of IFN-I, which needs to reach any potential target of viral infection.

However, cell-specific differences in the responsiveness to IFN-I signalling have been observed in humans, particularly among immune effector cells. Thus, IFN-I inducing TLR9 agonists, as well as HIV-1, efficiently upregulated the expression of the IFN-I regulated gene PDL1 on primary human monocytes and a subset of T lymphocytes, identified by selective expression of the chemokine receptor CCR5 [21]. Analysis of IFNAR2 expression in T lymphocytes revealed that this subunit of IFNAR was almost exclusively restricted to CCR5+ T cell subsets, independent of whether CD4 or CD8 T cells were considered [21]. A similar restriction of IFN-I signalling to CCR5+ T cells was observed when HIV-1-induced upregulation of the T cell activation markers CD38 and CD69 was analyzed *in vitro* [9, 140].

In both humans and non-human primates, CCR5 expression is generally higher in memory and effector T cell subsets [141–144], and natural disease-resistant SIV host species, such as sooty mangabeys, show reduced levels of CCR5+ CD4 T cells compared to disease susceptible hosts such as humans and Rhesus macaques [142, 143]. The role played by CCR5 as coreceptor for HIV-1 and SIV Env proteins, providing the critical mechanism for envelope-membrane fusion and injection of the viral RNA into the cytoplasm, provided the basis for investigating how differences in CCR5 expression between different species translate into different transmission and disease progression phenotype [142, 145]. However, little attention has been given to the possibility that different profiles of CCR5 expression among primate species may account for different sensitivity to IFN-I production, and therefore different patterns of regulation of IFN-I-stimulated genes during chronic HIV-1 or SIV infection, ultimately accounting for differences in disease susceptibility.

### 4.3. Cytostatic and Proapoptotic Effects

Type I IFN are widely recognized as the most potent natural mediators of antiviral activity in humans [1, 3]. Because the life cycle of viruses is directly influenced by the intracellular environment, efficient antiviral mechanisms have developed to interfere with basic cellular functions or, in an even more resolute manner, eliminate infected cells which represent the factories of viral replication. These goals are achieved by IFN-I via the upregulation of genes exerting cytostatic and proapoptotic activities. The broadly effective antiviral mechanisms include the induction of molecular mechanisms interfering with protein synthesis and cellular activity, as well as the stimulation of ligand/receptor-dependent apoptotic pathways and stress responses. While exerting potent antiretroviral activity, these mechanisms clearly interfere with basic cellular functions and carry a potentially destructive risk for the host, hence the need for tight regulation of both the kinetic of activation and the restriction to specific anatomical locations.

During HIV-1 infection, the chronic induction of IFN-I responses may contribute to progressive immunodeficiency [10, 146].

#### 4.3.1. Ligand-Receptor Mediated Apoptosis

Type I IFN positively regulates the expression of tumor necrosis factor (TNF) family members, capable of inducing apoptosis in cells which express specific receptors. Thus, expression of FAS ligand (FasL), TNF-related apoptosis inducing ligand (TRAIL) and programmed death ligand (PDL) 1, are all directly regulated by IFN-I signalling [21, 146–148].

The contribution of the Fas/FasL system to CD4 T cell apoptosis during HIV-1 infection is well documented [149–151], and similar findings were reported for IFN-I-induced TRAIL and its death receptor (DR)5 [55, 65, 121, 152, 153]. TRAIL expression following HIV-1 exposure or infection has been described in T cells, monocytes, and pDC [24, 55, 153, 154], and TRAIL expressing pDC have been shown to directly induce apoptosis of CD4 T cells in HIV-1 infected patients with high viraemia [153]. TRAIL mediated apoptosis in the setting of HIV-1 stimulation *in vitro* was originally reported to affect CD4 T cells, but not CD8 T cells [55, 65, 152]. Interestingly, TRAIL mediated CD4 T cell apoptosis did not require productive infection, and TRAIL expressing pDC were reported to be unable to lyse HIV-1 infected CD4 T cells [155] possibly favouring the depletion of uninfected CD4 T helper cells in the face of CD4 T cell viral reservoirs preservation. However, Zhu et al. later described TRAIL dependent apoptosis in HIV-1 infected macrophages, as a result of the downregulation of a decoy receptor [156]. Thus, TRIAL induced apoptosis may contribute to regulating the balance between clearance of HIV-1 infected cells and depletion of CD4 T helper cells.

Upregulation of PDL1 on circulating monocytes and T cells during HIV-1 infection was originally described to correlate with active viral replication [157]. The report of a causative link between IFN-I secretion and PDL1 expression after exposure to HIV-1 also indicated that CCR5 expression on T cells is associated with sensitivity to IFN-I signalling, determined by increased expression of IFNAR2 [21]. PDL1 engages its receptor PD1 on T lymphocytes to suppress proliferation and induce apoptosis [158]. During HIV-1 infection, PD1 is highly expressed by exhausted dysfunctional HIV-1-specific T cells [159], and blockade of PD1-PDL1 interaction enhances HIV-1-specific T cell immunity and immune-mediated control of viraemia in humanized mice and simian models of HIV-1 infection [160–164]. Thus, upregulation of PDL1 via IFN-I may directly contribute to the impairment of efficient antiviral T cell responses and to the perpetuation of HIV-1 infection.

#### 4.3.2. P53-Mediated Apoptosis

The existence of a functional connection between IFN-*α*/*β* and p53 we formally reported by Takaoka and coworkers in 2003 [165]. The relevance for IFN-I-induced p53 activation for viral infections was highlighted by Vilček, who postulated that IFN-I-mediated activation of the STAT1-STAT2-IRF-9 complex (ISGF-3) during viral infection may promote p53 gene expression via two ISRE sites in the p53 gene promoter [83].

Doitsh and colleagues reported that the accumulation of reverse transcription intermediates in CD4 T cells undergoing abortive HIV-1 infection results in apoptotic death [166]. The apoptotic pathway induced by abortive infection was associated with the production of inflammatory cytokines, including IL-1*β* and IFN-*β*, and involved activation of caspase-1 and caspase-3 [166]. However, the authors found no evidence of p53 involvement [166].

### 4.4. Viral Restriction Factors

HIV-1 infection and replication within infected cells can be inhibited at different steps of the viral life cycle by restriction factors regulated by IFN-I.

#### 4.4.1. Interferon Stimulated Gene 15 (ISG15)

The 15 kDa product of the IFN-stimulated gene (ISG) 15 was originally identified as a ubiquitin homologue^13^. Similar to ubiquitin, ISG15 acts as a tag for cellular proteins via covalent binding mediated by specific enzymes, which are also positively regulated by IFN-I [167–170]. However, different from ubiquitin, the primary effect of tagging with ISG15 (often referred to as ISGylation) does not appear to be proteasomal degradation. For example, ISGylation favours the sustained production of IFN-*β* in virus-infected cells by preventing the degradation of IRF3 [171] and enhances NF-*κ*B signalling by inhibiting the activity of the protein phosphatase 1B (PPM1B) [172].

ISG15 has been shown to exert broad antiviral activity in both mice and humans [167]. The anti-HIV-1 activity of ISG15 is mediated via inhibition of the ubiquitylation of the capside protein Gag and of the cellular protein encoded by the tumor susceptibility gene (Tsg)101 [173, 174]. Ubiquitylation of Gag and Tsg101 is required for the release of virions from infected cells, a process which is therefore inhibited by ISGylation [173, 174].

#### 4.4.2. Myxovirus Resistance (Mx) GTPases

Type I IFN induce the expression of proteins involved in membrane rearrangement leading to vesicle budding, organogenesis and cytokinesis. Some of these proteins may contribute to host resistance to pathogens, including the p47 and p65 guanylate-binding proteins (GBP), inducible GTPases and the myxovirus resistance (Mx) proteins [175]. In particular, the Mx proteins have been widely studied for their antiviral activity and their expression is strictly regulated by IFN-I [176].

Two Mx proteins have been identified in humans, MxA and MxB. MxA exerts inhibitory effect on a wide range of viruses [167]. MxB was recently described as a novel restriction factor for HIV-1 [177, 178]. The mechanism of HIV-1 inhibition is not fully elucidated, but the viral cycle is blocked after viral entry and before proviral DNA integration [177, 178]. In addition, binding to the capsid protein gag is required for the antiviral activity [177, 178].

#### 4.4.3. 2′-5′-Oligoadenylate Synthetase (OAS) and RNaseL Pathway

The 2′-5′-oligoadenylate synthetase (OAS) proteins are IFN-I inducible enzymes which polymerize ATP into oligomers of adenosine generated with noncanonical 2′-5′ bonds [179, 180]. These 2′-5′ adenosine oligomers induce activation of RNaseL, which mediates degradation of RNA from invading viruses and suppression of cellular activities [181]. Degraded RNA may also activate cytoplasmic RLR, resulting in IFN-I production and amplification of the antiviral innate responses [182].

Specific anti-HIV-1 activity by the OAS-RNaseL axis has been described in transfected cell lines [183]. Although the *in vivo* relevance of this antiviral system for HIV-1 inhibition is still to be determined, it is reasonable to speculate that the non virus specific effect of RNaseL may partially affect HIV-1 activity in target cells.

#### 4.4.4. Protein Kinase RNA-Activated (PKR)

The protein kinase RNA-activated (PKR) is involved, together with other members of the same family of protein kinases, in the response to environmental stress. Kinases in this family regulate the rate of protein synthesis by phosphorylating the *α*-subunit of the eukaryotic initiation factor (EIF) 2, which result in the sequestration of guanine-nucleotide exchange factor EIF2*β*, ultimately preventing the recycle of guanidin diphosphate (GDP) which is strictly necessary for protein synthesis [184].

PKR is constitutively expressed in all cell types in an inactive monomeric form and converts active homodimers following autophosphorylation at key residues [185–188]. The presence of two RNA-binding motifs in the N-terminal region allows for direct activation of PKR by RNA [189, 190].

PKR is a potent inhibitor of HIV-1 replication in human cell lines *in vitro*, but exerts lower inhibitory activity in primary cells [191–196], questioning the *in vivo* relevance of PKR-mediated anti-HIV-1 activity. HIV-1 messenger RNA contain a target sequence at the 5′ end which is recognized by the transactivating accessory protein Tat; the *trans*-activating response (TAR) RNA element plays a critical role in activating transcription [197–199]. PKR is sensitive to changes in the cytoplasmic concentration of TAR RNA sequences, in that low amounts of TAR induce PKR activation, whereas high TAR RNA concentrations exert an inhibitory effect on PKR activity [200–202].

#### 4.4.5. Tripartite Motif (TRIM) Proteins

Members of the tripartite motif (TRIM) protein family participate in a broad range of cellular functions, including apoptosis and antiviral immunity [203]. Several TRIM proteins have been described for their ability to restrict retroviruses replication and many are directly regulated by IFN-I in humans, including TRIM5 and TRIM22 [204–208].

TRIM5 is one of the best studied members of the TRIM family, mainly because of its inhibitory activity against HIV-1. TRIM5 blocks HIV-1 infection in the cytoplasm, before reverse transcription is completed, by recognizing and binding the capsid protein lattice, thus interfering with the uncoating and ultimately with reverse transcription [209]. The antiretroviral effect is strictly dependent on TRIM5 E3 ubiquitin ligase activity [210, 211], and is associated with proteasome recruitment [212, 213]. TRIM5 itself is ubiquitinated and degraded when it exert inhibitory activity on restriction-sensitive viruses [210, 211, 214, 215]. However, it is still unclear whether ubiquitination of HIV-1 capsid occurs. TRIM5 proteins from different nonhuman primate species show varying potency of anti-HIV-1 activity. The macaque and owl monkey TRIM5 orthologues have been particularly well studied, due to the potent inhibition of HIV-1 infection in cells from these species [216, 217], which is associated with strong interaction with the HIV-1 virion core [218]. The human TRIM5 orthologue shows relatively low inhibitory activity on lab-adapted HIV-1 stains [205, 219], but it is 10-fold more potent on certain primary HIV-1 isolates [220].

TRIM5 contributes to control viral transmission and replication in SIV-infected non human primates [221–225], and differences in the rate of disease progression in HIV-1 infected patients correlate with TRIM5 expression and polymorphisms [226–229]. In addition, Battivelli and colleagues reported that HIV-1 variants selected by cytotoxic T lymphocyte pressure to escape T cell recognition may be highly sensitive to restriction by human TRIM5 [230].

In addition to its well-studied antiviral activity, TRIM5 has been the subject of studies characterizing its role as a pattern recognition receptor (PRR) for the protein lattice of retroviral capsid. TRIM5 is required for dendritic cell activation in response to lipopolysaccharide (LPS) [211], and the signal transduction pathways involving AP-1 and NF-*κ*B are activated by TRIM5 [211, 231]. Thus, the interaction of TRIM5 with the HIV-1 capsid lattice enhances the induction of inflammatory cytokines [211].

Other TRIM proteins have been studied for their ability to interfere with HIV-1 replication. Human TRIM22 efficiently inhibits HIV-1 transcription and replication in primary human monocyte derived macrophages [232, 233]. TRIM22 exert its antiviral activity after HIV-1 integration, and E3 ubiquitin ligase activity is not necessary for TRIM22-mediated inhibition of HIV-1 replication [234]. Depending on the cell type and subcellular localization, TRIM22 can inhibit HIV-1 transcription at nuclear level [234] or virion production in the cytoplasm [204].

#### 4.4.6. Apolipoprotein B-Editing Catalytic Polypeptide 3 (APOBEC3) Proteins

The anti-HIV-1 activity of proteins of the apolipoprotein B-editing catalytic polypeptide 3 (APOBEC3) family was originally described in an effort to characterize the function of the HIV-1 accessory protein virion infectivity factor (Vif), which is required for HIV-1 replication in primary CD4^+^ T cells and certain cell lines, but not in fully permissive lines. Further studied demonstrated that the human gene APOBEC3G is a potent inhibitor of the replication of Vif-deficient HIV-1 [235].

The inhibition of HIV-1 replication by APOBEC3G (A3G) relies on its cytidine deaminase activity on both RNA and DNA, causing the conversion of cytidine residues into uridines [236–238]. This process results in both the alteration of the nucleotide sequence and the insertion of nonnatural bases (uredines) into the DNA strand [236–238]. The enzymatic activity of A3G is selective for the third cytidine in 5′-CCCA-3′ sequences [237, 238]. As a consequence of cytidine-to-uridine mutation, the levels of HIV-1 cDNA in infected cells are diminished. It was initially thought that uredine-containing DNA was degraded by host DNA repair enzymes, but inhibition of uracil DNA glycosidases is ineffective in restoring HIV-1 cDNA levels [239]. It has been suggested that A3G activity may interfere with the progression of the reverse transcriptase along the viral RNA template [240, 241].

One important characteristic of A3G is the fact that, unless counteracted by Vif, it is packaged into assembling HIV-1 virions [242]. Thus, A3G-bearing virions can transfer the restriction factor to target cells [237, 243–245].

The viral accessory protein Vif potently inhibits the antiviral activity of A3G by recruiting it for polyubiquitination and proteasomal degradation, thus preventing A3G incorporation into newly formed virions [244, 246–248].

Other human APOBEC proteins have been shown to exert anti-HIV-1 activity *in vitro*. However, only APOBEC3F (A3F) and APOBEC3H (A3H) haplotype II appear to have a significant effect *in vivo* [249], even though they appear to have lower potency and lower expression than A3G. Accordingly, A3G, A3F, and A3H are the only APOBEC3 proteins counteracted by Vif, suggesting a dynamic evolutionary interplay between host restriction factors and viral escape mechanisms.

#### 4.4.7. Bone Marrow Stromal Cell Antigen 2 (BST2, Tetherin)

The bone marrow stromal cell antigen 2 (BST2 or tetherin) is transmembrane protein with glycophosphatidylinositol lipid anchor at the C-terminal domain, an extracellular *α*-helix domain and a N-terminal transmembrane anchor [250]. Similar to A3G, tetherin was described as the IFN-I-induced cellular restriction factor inhibited by a HIV-1 accessory protein, namely Vpu [85, 86, 251, 252]. Thus, in the absence of Vpu, HIV-1 virions budding from productively infected cells are trapped or tethered (hence the name tetherin) on the surface of infected cells [85, 86, 251, 252]. Tethered virions are eventually internalization and accumulate in endosomes [251].

Tetherin exert antiviral activity on several families of viruses [253–255], and it preserves antiviral activity in the face of extensive mutations [256], suggesting that its function is not strictly dependent on recognition of specific viral protein sequences.

HIV-1 evades the antiviral effect of tetherin thanks to the accessory protein Vpu [86, 252]. Vpu directly binds tetherin, and causes a general decrease in tetherin expression on the cell surface [252]. Different mechanisms have been suggested to account for Vpu-mediated counteracting of tetherin activity, including alterations of trafficking pathways [257, 258], and enhanced proteasomal degradation [259–261].

Other human and nonhuman primate lentiviruses have developed mechanisms of evasion for tetherin-mediated antiviral activity. Thus, most SIV do not encode Vpu, and tetherin is antagonized by the Nef protein [262, 263]. Conversely, the Env protein of HIV-2, which also does not encode Vpu, has evolved to interfere with tetherin and cause it to be sequestered within intracellular compartments [264, 265].

#### 4.4.8. Sterile *α* Motif (SAM) and Histidine-Aspartic (HD) Domain-Containing Protein 1 (SMAHD1)

Studies on the function of another lentiviral accessory protein, the viral protein x (Vpx), brought to the discovery of the restriction factor sterile *α* motif (SAM) and histidine-aspartic (HD) domain-containing protein 1 (SMAHD1).

Vpx is expressed by HIV-2 and related viruses (such as SIVsm) but not by HIV-1 [266]. HIV-1 infection of human monocyte-derived dendritic cells is enhanced in presence of exogenous Vpx or if HIV-1 is genetically modified to encode for Vpx [267, 268], and HIV-2 reverse transcription in monocyte-derived macrophages is strictly dependent on Vpx [269]. A number of studies contributed to identify SAMHD1 as the target of Vpx-driven ubiquitylation and subsequent proteosomal degradation. Thus, SAMHD1 ubiquitylation is mediated by the cullin-4A ubiquitin ligase (CUL4A)-DNA damage-binding protein 1 (DDB1) complex (CUL4A-DDB1), which is assembled by Vpx via recruitment of the DDB1 CUL4A associated factor 1 (DCAF1) [270–273].

SAMHD1 exerts phosphohydrolase activity, and can degrade deoxyribonucleotides triphosphate (dNTP) to deoxynucleoside and inorganic triphosphate [274]. Reverse transcription relies on the availability of dNTP, and the anti-HIV-1 effect of SAMHD1 is mediated via its dNTPase activity, which causes a reduction of the pool of intracellular dNTP to a level incompatible with viral replication [275]. SAMHD1 is exclusively localized in the nucleus [276–278], whereas reverse transcription occurs in large part in the cytoplasm, suggesting that dNTP hydrolysis in the nucleus has deleterious repercussions on the cytoplasmic availability of dNTP.

SAMHD1 is expressed at constitutively high levels by cells which are nonpermissive with regards to HIV-1 infection, such as monocytes and monocyte-derived DC [271]. However, HEK293T cells, undifferentiated THP-1 cells and activated CD4^+^ T cells are not refractory to HIV-1 infection despite expressing SAMHD1 [270, 275], suggesting that the antiviral activity of SAMHD1 may be biologically important only in differentiated or non-dividing cells such as DC and macrophages. The high rate of dNTP turnover in dividing cells, overcoming the dNTPase activity, may in part explain the susceptibility to lentiviral infection despite SAMHD1 expression. SAMHD1 can be upregulated in human monocytes and monocyte-derived DC by either type I or type II IFN [279, 280].

### 4.5. Immunostimulatory and Modulatory Activites

Type I IFN play a pivotal role in the transition from innate to adaptive immune responses by both stimulating maturation of APC and by directly modulating and shaping T cell function.

#### 4.5.1. Effect of IFN-I on APC Differentiation and Maturation

IFN-I stimulates monocytes differentiation into DC *in vitro* [281–284]. However, the presence of IL-4 in the *in vitro* culture of monocyte appears to modify the effects of IFN-I, likely due to the ability of IL-4 to attenuate the expression of ISG [285]. Thus, the ultimate effect of IFN-I stimulation on the differentiation of monocytes into DC may depend on the cytokine milieu in the surrounding environment and the timing of exposure to different stimuli.

Importantly, IFN-I promotes the maturation of DC to fully competent APC by inducing upregulation of the costimulatory molecules CD40, CD80, and CD86 and by enhancing the expression of MHC class I and II [6, 7, 286, 287]. These changes in DC phenotype correspond to increased ability of IFN-I-stimulated DC to stimulate T cell proliferation *in vitro* [6, 7, 286, 287].

Following IFN-I stimulation, DC secrete IL-15, which exert stimulatory effects on T cells and promotes DC maturation [282, 288]. In addition, IL-10 and the TNF family members B lymphocyte stimulator protein (BlyS) and APRIL are also expressed by DC following IFN-I stimulation [289–291]. When expressed in combination with IL-10 or transforming growth factor (TGF)*β*, APRIL can induce T cell independent immunoglobulin class switching [291]. IL-10 is a potent anti-inflammatory mediator, its production in response to IFN-I highlights the dichotomous role that IFN-I may play in balancing immune activation and immune suppression [289]. Accordingly, the effect of IFN-*α*/*β* on IL-12 production can be either stimulatory or inhibitory [292–294]. The maturation stage of DC and the dose of IFN-I to which they are exposed may all affect IL-12 production, as well as the interaction of IFN-I with other stimuli [295, 296]. Changes in the secretion of IL-12 may then affect the quality of the DC-activated T cell response by modulating the induction of IFN-*γ*-secreting cells as well as the production of other cytokines, such as IL-4, IL-5, IL-10, and IL-13 [289].

The secretion of chemokine is also modulated by IFN-I. Monocyte-derived immature DC secrete the CXCR3-binding chemokines CXCL9, CXCL11, and CXCL10 in response to IFN-I stimulation [287]. CXCR3 is expressed by activated T and B cells [297], which may be recruited to sites of inflammation. In addition, IFN-I induces DC-mediated secretion of CXCL19 (MIP-3*β*), which recruits naive T cells and may therefore contribute to initiating adaptive T cell responses [298].

#### 4.5.2. Modulation of T Cell Response

The direct effect of IFN-I on proapoptotic and immunoregulatory ligands, such as FasL, TRAIL, and PDL1, has been discussed in Section 4.3.

The influence that IFN-I exert on APC maturation and cytokine production underscore the key role played in the differentiation of both CD4 and CD8 T cells. Thus, the enhancement of IL-12 production observed when DC are matured in presence of IFN-I, induces STAT4 activation and subsequent T-bet expression in CD4 T cells, which drive the differentiation into IFN-*γ*-producing T helper (Th)1 cells [299]. In addition, IFN-I directly act on human T cells to induce STAT4 activation [300, 301], which is however not sufficient on its own to drive Th1 commitment *in vitro* [302, 303]. Conversely, in presence of other cytokines, such as IL-18 and IL-21, IFN-I efficiently promotes Th1 cell differentiation and effector functions [304–306].

In parallel to the positive effects on Th1 differentiation, IFN-I may inhibit or reverse CD4 T cell differentiation into other Th types. Thus, Th2 responses are negatively regulated by IFN-I in human cells via the suppression of the Th2-driving transcription GATA-binding protein 3 (GATA3) [304]. Furthermore, suppression of Th17 differentiation by IFN-I has been described in both murine and human cells [307, 308].

IFN-I may also contribute to negatively modulate T cell response by regulating the function of CD4 regulatory T cells (Treg), which play a critical role in the maintenance of immunological tolerance and homeostasis. Studies conducted in patients with relapsing-remitting multiple sclerosis have highlighted how administration of IFN-*β* treatment enhanced both the frequency and suppressive function of *T*_Reg_ cells [130].

The primary response of CD8 T cell against viral infection, epitomized by proliferation and clonal expansion, can be enhanced in presence of IFN-I, which is also critical in allowing the generation of memory CD8 T cells [309–311]. However, secondary CD8 and CD4 T cell responses against viral antigens, including HIV-1, may be inhibited in presence of high levels of IFN-I [9, 21]. Thus, the effect of IFN-I on T cell responses may largely depend on the stimulation condition and the cell types affected, in that IFN-I may favour costimulatory signals during primary antigen-specific responses by naive T cells, but suppress secondary memory responses against recall antigens via the induction of cytostatic and proapoptotic mechanisms.

## 5. Conclusion and New Perspectives

Type I IFN play a central role in the coordination of a multitude of immune effector and regulatory mechanisms. The ultimate effect of IFN-I on the overall economy of an effective antiviral immune response may depend on the mechanism of induction, the cellular source, and the timing of production.

Plasmacytoid DC may respond rapidly to viral exposure. The ability of pDC to secrete IFN-I in response TLR7/9 signalling indicates that these cells do not require the viral genome to enter the cytoplasm, meaning that they produce IFN-I independent of whether they are productively infected (Section 2.1). Combining IFN-I production with the potential to mature into APC, pDC may be the ideal candidate to promote efficient antiviral primary T cell responses, by favouring CD4 T cell differentiation into Th1 cells and favouring the activation and clonal expansion of virus-specific naive CD8 T cells (Sections 3.1 and 4.5).The local production of IFN-I at the site of infection by pDC also favours the induction of antiviral mechanisms which may limit the spread of infection (Section 4.4).

When productive infection of some tissue target cells is established, IFN-I production may be sustained by infected cells which do not normally exert immune function, but can react to viral genomes in the cytoplasm via specific sensors (Sections 2.2 and 2.3). Although limited in terms of amount of IFN-I produced on a per cell basis, this second pDC-independent wave of IFN-I production may be more focused and circumscribed to the foci of potential viral replication, and efficiently inhibit virus spread to other cells without interfering with adaptive immune responses.

During HIV-1 infection, both the early pDC-mediated and the late pDC-independent IFN-I responses may be dysfunctional and cause severe damage to the development and maintenance of adaptive anti-HIV-1 responses. Thus, the organization of the HIV-1 envelope into a functional lipid raft may favour an overwhelming induction of pDC-mediated persistent IFN-I responses over antigen-presenting function (Section 3.1), which may prevent the efficient promotion of primary CD4 Th1 and CD8 T cell responses. Subsequently, the tropism of HIV-1 for immune cells, including DC and monocyte/macrophages, may result in the prolonged production of IFN-I in lymphoid tissues by HIV-1-infected professional APC, which may compromise the induction of secondary HIV-1-specific responses and contribute to the perpetuation of infection and disease progression.
